# A critical study on efficiency of different materials for fluoride removal from aqueous media

**DOI:** 10.1186/1752-153X-7-51

**Published:** 2013-03-13

**Authors:** Vaishali Tomar, Dinesh Kumar

**Affiliations:** 1 Department of Chemistry, Banasthali Vidyapith, Rajasthan, 304022, India

**Keywords:** Adsorbents, Adsorption, Fluoride, Removal

## Abstract

Fluoride is a persistent and non-biodegradable pollutant that accumulates in soil, plants, wildlife and in human beings. Therefore, knowledge of its removal, using best technique with optimum efficiency is needed. The present survey highlights on efficacy of different materials for the removal of fluoride from water. The most important results of extensive studies on various key factors (pH, agitation time, initial fluoride concentration, temperature, particle size, surface area, presence and nature of counter ions and solvent dose) fluctuate fluoride removal capacity of materials are reviewed.

## Introduction

The chemical nature of water is one of the most imperative criteria that determine its usefulness for a precise need and as such not all the waters are fit for drinking and potable purposes. Apart from fluoride, arsenic and nitrate are few of major water pollutants which cause large scale health issues, but in cutting-edge the most serious pollutant is fluoride [1]. According to the World Health Organization the maximum acceptable concentration of fluoride ions in drinking water lies below 1.5 ppm. Fluoride if taken in small amount is usually beneficial, but the beneficial fluoride concentration range for human health is very small. Depending on the concentrations and the duration of fluoride intake, it could have positive effect on dental caries [2]. On the contrary, long term consumption of water containing excessive amounts of fluoride can lead to fluorosis of the teeth and bones [3]. The excessive intake of fluoride may cause dental [4] and skeletal disorders [5]. Fluoride ion is attracted by positively charged calcium ion in teeth and bones due to its strong electronegativity which results in dental, skeletal and no skeletal forms of fluorosis i.e. high fluoride ingestion, in children as well as adults. Fluorosis in mild version can be evidenced by mottling of teeth and in high version by embrittlement of bones and neurological damage [6], in some of the cases it may even interfere with carbohydrates, proteins, vitamins and mineral metabolism and to DNA creation as well if intake excessively [7]. Studies have shown that major of the Kidney diseases have a great inclination of toxicity of fluoride. On high doses and short term exposure fluoride can exterminate the kidney function. Several research groups have also shows that fluoride can interfere with the function of pineal gland as well as of brain. Pineal gland is one of the major fluoride accrued site in body with concentration more than that of teeth and bones. Workers exposed to high fluoride concentration areas are diagnosed with bladder cancer [8]. Various diseases such as osteoporosis, arthritis, brittle bones, cancer, infertility, brain damage, Alzheimer syndrome, and thyroid disorder can attack human body on excessive intake of fluoride [9]. Fluoride contamination in ground water is a world-wide issue, and some cost effective technologies are required to eliminate excess fluoride in water. The occurrence of high fluoride concentrations in groundwater and risk of fluorosis associated with using such water for human consumption is a problem faced by many countries, notably India, Sri Lanka, and China, the Rift Valley countries in East Africa, Turkey, and parts of South Africa. Conventionally, the fluoride was removed from contaminated water is by liming and accompanying precipitation of fluoride [10]. Various other methods used for the defluoridation of water are ion-exchange [11], precipitation with iron (III) [12], activated alumina [13], alum sludge [14], calcium [15] is widely examined. In addition reverse osmosis [16,17] and electro coagulation [18]. Many of these methods didn’t get used on large scale because various unfavourable factors such as high operational and maintenance cost, generation of toxic by-products (pollution) and due to complex treatment. Authors discussed pros and cons of different techniques for defluoridation and it was concluded that the effective method is coagulation but it does not help in bringing down the fluoride concentration at desired level. On the other hand membrane process is expensive in terms of installation and operation cost, there are also more chances of fouling, scaling or membrane degradation. The electrochemical techniques are not popular due to high cost during installation and maintenance.

One of the most popular techniques for defluoridation that is used in countries like India, Kenya, Senegal and Tanzania is Nalgonda technique. In this technique, calculated quantities of alum, lime and bleaching powder are mixed with water, after mixing the water is processed with flocculation, sedimentation, filtration and disinfection. The entire operation takes about 2–3 hours for around 200 people in batches. Disadvantages of this technique is reported that treated water has high residual aluminium concentration (2–7 mg/L) then the WHO standard of 0.2 mg/L [19-21]. Among these methods, adsorption is the most suitable and widely used technique due to its simple operation, and the availability of a wide range of adsorbents [22].

In this review, an extensive list of adsorbents literature has been compiled. It is evident from a literature survey of about 140 recent papers that low-cost sorbents have demonstrated outstanding removal capabilities for fluoride. In particular, nanomaterial based adsorbents might be a promising adsorbents for environmental and purification purposes.

## Review

### Remediation materials

#### Alumina and aluminium

Among the adsorbents, activated alumina has been an effective adsorbent for defluoridation of water. Different studies have been focus on this. For the fluoride removal from water Acidic alumina [23], amorphous Al(OH)_3_, gibbsite or alumina (Al_2_O_3_) [24] have been used. It was found that this adsorbent react fluoride at pH range 3–8 with fluoride concentration 1.9 -19 mg/L. At pH 5.5-6.5, maximum fluoride uptake was observed 9 mol/kg. At lower pH, fluoride uptake decreased due to the preferential formation of AlF_x_ soluble species but at higher pH, OH^−^ displaced F^−^ from the solid Al(OH)_3_ so the amount of fluoride adsorbed to complexes declined towards zero between pH 6–8. At lower rate, same reaction was followed with gibbsite. At pH 5–7, maximum fluoride removal was found 16.3 mg/g. Due to the electrostatic repulsion in acidic solutions, adsorption of fluoride was retarded. At higher pH, fluoride adsorption on alum occurred due to electrostatic repulsion of fluoride ion to the negatively charged surface of alumina; competition for active sites by excusive amount of hydroxide ion [25]. The Langmuir and Freundlich isotherm models have been described the equilibrium behaviours of the adsorption process. So it was conclude that defluoridation by alumina occurred by nonspecific adsorption. The value of zeta potential for α- Al_2_O_3_ was also investigated_._ At 25°C and pH 5–6, maximum fluoride removal was occurred. From the zeta potential measurement, it was achieved that fluoride adsorbed onto α- Al_2_O_3_ by replacing hydroxyl ions from positively charged surfaces and through hydrogen bond [26]. Adsorption capacity of activated alumina (AA) (grad OA −25) was also studied for defluoridation of aqueous solution. At pH 7, adsorption capacity was obtained 1450 mg/kg [27]. Defluoridation increased at pH 4–7 but decreased thereafter. At pH >7, silicates and hydroxyl ions were considered to compete with F^−^ ions for alumina exchange sites but at pH < 7, alumina fluoro complexes were formed in the presence of aluminium ions in the treated water. The potential of metallurgical grade alumina (MGA) for defluoridation was investigated at different condition [28]. The effect of temperature on zeta potential and fluoride adsorption was observed at α- Al_2_O_3_/aqueous solution interface [29]. Comparison between the adsorption capacities of the untreated hydrated alumina (UHA) and thermally treated hydrated alumina (THA) were obtained from the hydrolysis of locally manufactured aluminium sulphate for defluoridation [30]. The capacity was found 23.7 mg F^−^/g and 7.0 mg F^−^/g for THA and UHA, respectively at pH 4–9. The potential of alumina for fluoride removal from aqueous solution was explained by several other researchers [31-34].

In the recent study, the application of the new HPLC–UVVIS method used in speciation analysis of aluminum form Al(III) ion, aluminum complexes with fluorides and iron in groundwater samples. Based on the obtained results of groundwater samples analysis, the separation of iron in the retention time ≈ 3.7, was obtained. The proposed method is selective for aluminum fluoride complexes and Al(III) in the pH conditions of their occurrence. The paper presents the possible types of transformation of aluminum hydroxyl forms and aluminum sulfate complexes by the reaction of the sample with mobile phase. An indirect method for the determination of aluminum in the form of aluminum sulfate was proposed [35]. See Table 1 for details.

**Table 1:** **Adsorption capacities** (**AC**) **and other parameters for the removal of fluoride by Alumina and aluminium**

**Adsorbent**	**AC (mg/g)**	**CR (mg/L)**	**pH**	**Ref.**
Acidic alumina	8.4	5-15	3.6-11.6	[23]
Activated alumina (α- Al_2_O_3_)	88	15-100	5-6	[26]
Activated alumina (Grade OA-25)	2	2.5-14	7	[27]
Metallurgical grade alumina	13	-	5-6	[28]
Al(OH)_3_ (Al_2_O_3_.xH_2_O) (THA and UHA)	24 and 7	5-30	7 ± 0.3	[30]

#### Modified activated alumina

To improve the adsorption capacity of alumina, it has been modified. For the modification of it, it has been impregnated by La(III) and Y(III) [36]. Comparison was witnessed between La(III) and Y(III) impregnated alumina and original alumina for the adsorption of fluoride. Lanthanum hydroxide supported on alumina has also been investigated foe defluoridation [37]. The adsorption capacity of alumina impregnated lanthanum hydroxide was 48 mg/g, which was higher than original alumina 23–25 mg/g. Defluoridation by impregnated alumina was explained by ion exchange process between anion and hydroxide group on surface material. Adsorption was found to increase with decreasing of fluoride concentration from 130 mg/L to 0.57 mg/L at pH 5.7-8.0. The sorbed fluoride ions were eluted from the adsorbent with 10 mL of 0.1 M NaOH and column could be reused after being conditioned with 10 mL of 1 × 10^−3^ M HCl. The surface of alumina was also impregnated with alum for defluoridation [38]. At pH 6.5, the adsorption of fluoride was found 92.6% and then decreased with further increased in pH. To improve the efficiency of alumina for defluoridation of aqueous solution, it modified by coating of copper oxide. The adsorption capacity of copper oxide coated alumina was mentioned in Table 2, which was higher than unmodified activated alumina [39]. Magnesia amended activated alumina [40,41] and calcium oxide modified activated alumina [42] was also used for fluoride removal from water. See Table 2 for details.

**Table 2:** **Adsorption capacities** (**AC**) **and other parameters for the removal of fluoride by modified activated alumina**

**Adsorbent**	**AC (mg/g)**	**CR (mg/L)**	**pH**	**Ref.**
La(III) impregnated on alumina	48.0	276 -300	5.7-8.0	[37]
Alum-impregnated activated alumina	40.7	1-35	6.5	[38]
Copper oxide coated alumina	7.8	10- 15	-	[39]
Magnesia-amended activated alumina	10.1	5-150	6.5-7.0	[40]
Calcium oxide-modified activated alumina	101.0	1-1000	5.5	[42]

#### Iron-based adsorbents

Iron-based materials have been investigated for fluoride removal from water. Polypyrrole (PPy)/Fe_3_O_4_ magnetic nanocomposites is novel adsorbent in fluoride removal [43]. It was found that presence of chloride and nitrate ions had negligible effect on fluoride adsorption while sulphate and phosphate ions reduced fluoride adsorption because of competitive interaction. It was found that fluoride, sulphate, phosphate ions form inner sphere complexes and they competed for the same active sites of adsorption. Fluoride removal found to be increased with increase in polypyrrole/magnetite (adsorbent) dose. Fluoride uptake increased with increase in solution pH from 2 to 6. Up to 97% of the adsorbed fluoride on the PPy/Fe_3_O_4_ nanocomposites was desorbed at pH 12. The adsorption process was endothermic in nature and proceeded by ion-exchange mechanism. To understand the mechanism of fluoride removal, electro coagulation is a method of applying direct current to sacrificial electrodes that [44] are submerged in an aqueous solution and in the acidic condition dissolving aluminum Al(III) is predominant and aluminum hydroxide has tendency soluble. And electro coagulation is pH dependent and pH ranging from 6 to 8, the defluoridation process was found to be efficient. The fluoride removal mechanisms were investigated based on the solution speciation (Al and Al–Fe complexes) and dried sludge characteristics in the electro coagulator. Fluoride removal by amorphous Fe/Al mixed hydroxides was evaluated [45]. At pH 7.5, mixed Fe/Al samples were prepared by the increase of Al content in Fe(OH)_3_ matrix increase the surface area. The fluoride adsorption followed first order kinetics and intraparticle diffusion model. The sorption process followed both Langmuir and Freundlich isotherm models. The thermodynamic studies showed fluoride sorption to be spontaneous and exothermic in nature. Adsorption and desorption studies were also conducted to gain an insight into the adsorption mechanism on Fe/Al hydroxide surface. The formation of new complexes on the fluoride adsorbed on adsorbent surface was confirmed through spectral analyses. The other anions like phosphate, sulphate and arsenate concentration have adverse effect on fluoride removal efficiency of adsorbent. The adsorbent regenerated with 0.5 M NaOH, maximum regeneration of 80.5% was obtained.

Granulated mixture of Fe-Al-Ce nano adsorbent for fluoride removal by spraying Fe-Al-Ce nano-adsorbent suspension onto glass beads in a fluidized bed was developed [46]. It was found that fluoride adsorption capacity was 2.22 mg/g at pH 7 and initial fluoride concentration of 1 × 10^−3^ M using the coated granules as adsorbent. The active site for fluoride adsorption was the hydroxyl groups on the Fe-Al-Ce surface. With the increasing coating amount the adsorption capacity increased while the stability of the granule decreased. The natural stilbite zeolite modified with Fe(III) used as adsorbent for the removal of excess fluoride from the drinking water [47]. It was studied from the batch adsorption studies that fluoride concentration can be reduced down to the very low level of 1 mg/L under the optimum conditions. From the study of XPS and EDX, it was concluded that Fe(III) is impregnated on the natural stilbite zeolite and the fluoride is adsorbed on the Fe(III)-stilbite zeolite. And the result of desorption and regeneration showed that the exhausted Fe(III)-stilbite zeolite can be regenerated using 1 M HCl as eluent and the regenerated samples still remain the good adsorptive performance. The fluoride adsorption on Fe(III)-STI is well described by the Langmuir adsorption model, and the maximum adsorption capacity is 2.31 mg/g. The natural STI zeolite is an environment-friendly adsorbent with lower chemical consumption and waste release and operating costs.

For the effectiveness of iron-impregnated granules ceramics in defluoridation of water, it was prepared by simple granulation procedure at room temperature. Both granular ceramics FeSO_4_.7H_2_O and granular ceramic (Fe_2_O_3_) adsorbents was used for defluoridation of aqueous solution [48]. It was found granular ceramics FeSO_4_.7H_2_O is more effective than granular ceramic (Fe_2_O_3_) for fluoride removal. The adsorption experiments by batch and mini column scale to test the potential of granular ferric hydroxide for removal of various ions including fluoride was studied [49]. The fluoride adsorption onto granular ferric hydroxide was again found pH dependent. The fluoride capacity decreased with increasing value of pH. The fluoride has the highest adsorption capacity (1.8 mmol/g) and it followed by arsenate (0.9 − 1.0 mmol/g) and phosphate (0.65 − 0.75 mmol/g). The decreases were sharp at above pH 8, as the surface charge of the sample became more negative. The fluoride did not affect arsenate uptake by the adsorbent as fluoride is not a triprotic acid and does not compete for the same sites as arsenate. The synthetic siderite used as a sorbent for fluoride removal [50]. In experiments with an adsorbent dosage of 5 g/L, which was up to 1.775 mg/g and an initial F^−^ concentration was 20 mg/L at 25°C. The presence of chloride and nitrate has less effect on fluoride adsorption, while phosphate ion has affected F − removal capacity from aqueous solution. Co-precipitation of ferric hydroxide with fluoride was caused by dissolution of pristine synthetic siderite and subsequent oxidization of Fe(II) ions. A novel bimetallic oxide adsorbent was synthesized by the co-precipitation of Fe(II) and Ti(IV) sulphate solution using ammonia titration at room temperature for fluoride removal from water [51]. Mg-doped nano ferrihydrite powder [52], Fe(III) modified montmorillonite [53], iron rich laterite [54], as an adsorbents for F^−^ removal from aqueous solutions. See Table 3 for details.

**Table 3:** **Adsorption capacities** (**AC**) **and other parameters for the removal of fluoride by Iron**-**based adsorbents**

**Adsorbent**	**AC (mg/g)**	**AD (g/L)**	**CR (mg/L)**	**pH**	**Ref.**
PPy/Fe_3_O_4_ nanocomposite	17.6 − 22.3	2.0	5-100	6.5	[43]
Fe/Al hydroxide	91.7	0.5	10-90	4.0	[45]
Fe-Al-Ce nanoadsorbent	2.22	5.0	19-25	7.0	[46]
Stilbite zeolite modified with Fe(III)	2.31	10.0	5-40	6.9	[47]
GC(FeSO_4_.7H_2_O), GC(Fe_2_O_3_)	2.16, 1.70	20.0	5-50	6.9	[48]
Synthetic Siderite	1.77	4-40	3-20	4-9	[50]
Fe―Ti oxide nano-adsorbent	47.0	0.5	50	6.9	[51]
Mg-doped nano ferrihydrite powder	64.0	0.5-4	10-150	5.75	[52]
Fe(III) modified montmorillonite	-	1	2-10	3.5-9.0	[53]

#### Calcium-based adsorbents

Calcium has a good affinity for fluoride anion and it has been used for fluoride removal [55]. Crushed limestone (99% pure calcite) used as an adsorbent for fluoride removal by batch studies and surface-sensitive techniques from solutions with high fluoride concentration ranging from 3 to ~2100 mg/L. With different techniques, such as atomic force microscopy (AFM) and X-ray photoelectron spectroscopy (XPS) as well as ζ potential measurements, the authors were able to confirm that a combination of surface adsorption and precipitation reactions removed fluoride from aqueous systems. The removal capacity of fluoride was dependent on calcite surface area. Activated and ordinary quick lime as adsorbents used for fluoride removal from water [56]. When initial concentration was 50 mg/L, the removal of fluoride was 80.6% at optimum conditions from synthetic solution. Langmuir maximum sorption capacity of activated quick lime for fluoride was found 16.67 mg/g. The removal of fluoride was found due to chemisorption and precipitation which was confirmed through scanning electron microscopy (SEM) micrographs and X-ray diffraction (XRD). Aluminium hydroxide impregnated limestone as a adsorbent used for fluoride removal from water [57]. At pH 2, the adsorption in case of modified lime stone was decreased. The maximum sorption capacities of the limestone and aluminium hydroxide impregnated limestone were found 43.10 mg/g and 84.03 mg/g, respectively. The adsorption method was used for fluoride removal from aqueous solution by Apatitic tricalcium phosphate [58]. The fluoride uptake by various calcium phosphate minerals [59], calcium aluminate (CA) [60] was reported. See Table 4 for details.

**Table 4:** **Adsorption capacities** (**AC**) **and other parameters for the removal of fluoride by Calcium**-**based adsorbents**

**Adsorbent**	**AC (mg/g)**	**CR (mg/L)**	**pH**	**Ref.**
Quick Lime	16.67	10-50	-	[56]
Lime stone & Al(OH)_3_ impregnated lime stone	43.10 & 84.03	0-100	8	[57]
Apatitic tricalcium phosphate	15.15	30-60	6.8-6.9	[58]
Calcium Aluminate (CA)	4.37	8.9	3-11	[60]

#### Other metal oxides/hydroxides/oxyhydroxides, mixed metal oxides, metal- impregnated oxides as adsorbents

An inorganic cerium based adsorbent used for fluoride removal [61] and showed sorption capacity for fluoride. Fluoride removal results at low pH were very fruitful. In the fluoride removal, hydroxyl group of cerium based adsorbent played vital role. To facilitate the adsorption of different cations and anions, the metal oxyhydroxide have surface oxygen which differs in the number of coordinating metal ions and the property of oxide minerals was found as advantages for fluoride removal from water [62]. Refractory grade bauxite feed bauxite, manganese ore and hydrated oxides of manganese ores used as adsorbents for fluoride removal from water. Experiments showed that refractory grade bauxite had high fluoride removal efficiency in comparison to other three adsorbents. With pH up to 5.5, the percentage of fluoride adsorption onto refractory grade bauxite was found to increase but decreased with increase in pH. The adsorption process was found to be exothermic hence the adsorption efficiencies decreased with increase of temperature. To obtain CeO_2_-TiO_2_/SiO_2_ surface, sol–gel method was employed and used to determine its potential for fluoride removal [63]. The adsorption capacity for fluoride was found 21.4 mg/g. Magnesia-amended silicon dioxide granules for fluoride removal were investigated [64]. With magnesium chloride solution, the modification of silicon dioxide by wet impregnation was also performed. The adsorption of fluoride depends upon the porous structure and high surface area of the modified granules. At pH range 3 to 4, maximum fluoride adsorption was found and further decreased as pH increased above 10 and the decreased in defluoridation was found due to the change in surface charge of the adsorbent. At pH 3, maximum defluoridation capacity was found 12.6 mg/g. The order of the reduction of fluoride adsorption is bicarbonate > sulphate > phosphate.

The reduction of fluoride adsorption was found due to the competition for active sites between these ions or due to the change in pH. Nano-sized superparamagnetic zirconia material (ZrO_2_/SiO_2_/Fe_3_O_4_, SPMZ) was applied for the sorption of fluoride from water and simulated industrial wastewater [65]. Fluoride removal from water by a mechanochemically synthesized anion clay (meixnerite) and its calcinations product was studied at initial fluoride:meixnerite molar ratios (FI:meix) of 0.1 to 2.0 the theoretical fluoride uptake limit for meixnerite [66].

Al-Ce hybrid adsorbent by co-precipitation method was prepared and used for fluoride removal [67]. The hybrid adsorbent was of amorphous structure with some aggregated nanoparticles which was revealed by SEM and XRD results. For fluoride, the adsorbent capacity of Al-Ce adsorbent was 91.4 mg/g at 25°C. At pH 6, maximum adsorption capacity was achieved. Due to high zero point potential, the adsorbent was effective in fluoride removal from aqueous solution. FTIR analysis and zeta potential measurement confirmed that the hydroxyl and pronated hydroxyl groups on the adsorbent surface were involved in fluoride adsorption at high and low pH solutions, respectively. Gel like titanium hydroxide derived adsorbent from titanium oxysulfate TiO(SO_4_) used for fluoride removal [68]. At low fluoride concentration the adsorbent exhibited high adsorption potential for fluoride and had selectivity for fluoride ions with coexisting chloride, nitrate and sulphate ions. At pH 3, maximum fluoride adsorption occurred. In case of low fluoride concentration < 0.8 mg/L, the adsorbent was also able to remove fluoride in real waste water. Aluminium titanate (AT) and bismuth aluminate (BA) as a adsorbent [69] used by authors due to high refractivity, low thermal conductivity, low thermal expansion coefficient of aluminium titanate and bismuth aluminate is antacid, nontoxic, water insoluble material which make them suitable for fluoride removal from water. The amounts of fluoride adsorbed by AT and BA were 0.85 and 1.55 mg/g, respectively at 30°C from 4 mg/L initial concentration.

The removal of fluoride from aqueous solution with magnesia (MgO) and magnesia/chitosan (MgOC) composite were used batch equilibrium experiments [70]. It was observed that defluoridation capacity of MgOC composite (4440 mg/F^−^/kg) was appreciably higher than MgO (2175 mg/F^−^/kg). The influence of different parameters such as contact time, co-existing anions and initial fluoride concentration were studied. It was found that MgO composite reached saturation after 30 min while MgO attained equilibrium after 60 min. In the presence of Cl^−^, ${\mathrm{SO}}_{4}^{2-}$ and ${\mathrm{NO}}_{3}^{-}$ ions the defluoridation capacity of MgOC composite was slightly increased while it decreased in presence of ${\mathrm{HCO}}_{3}^{-}$ ions. Defluoridation capacity of both MgO and MgOC composites were found to increase with increase in initial fluoride concentration. The sorption process followed Freundlich isotherm and pseudo-second order kinetics. The mechanism of fluoride removal was mainly governed by adsorption. Thermodynamics parameters (ΔGº, ΔHº and ΔSº) were calculate and values indicate that fluoride adsorption to be feasible, spontaneous and endothermic reaction.

The removal of fluoride by novel adsorbent calcined magnesia with pullulan (cMgOP) composite, an extracellular water soluble microbial polysaccharide was studied [71]. It was found that the surface area and adsorption micropore of the cMgOP composite were increased from 7.6 m^2^/g and 14 nm for pure MgO to 33 m^2^/g and 30 nm, respectively. The presence of Cl^−^, ${\mathrm{SO}}_{4}^{2-}$ and ${\mathrm{NO}}_{3}^{-}$ ions has negligible effect in defluoridation capacity because Cl^−^, ${\mathrm{SO}}_{4}^{2-}$ and ${\mathrm{NO}}_{3}^{-}$ ions interfered in fluorine reagent spectrophotometry and showed a small positive effect as to be negligible while significant decrease in presence of ${\mathrm{HCO}}_{3}^{-}$ due to the competition of bicarbonate ions with fluoride ions in the sorption process. Effect of contact time revealed that the adsorption of three adsorbents- cMgOP, MgO and pullulan increased with increasing contact time. The defluoridation capability of cMgOP (4537 mg/F^−^/kg) was 10 times than that of MgO (457 mg/F^−^/kg). At pH 5, maximum defluoridation capacity of 97.6% was obtained. From the thermodynamic parameters, sorption process was found to be spontaneous and endothermic. Particle diffusion model was the best to describe the adsorption of fluoride on cMgOP.

Hybrid process was applied that combined the adsorption on conventional solid adsorbents such as aluminium and zirconium oxide along with specific donnan dialysis for defluoridation of ground water [72]. It was found that adsorption was not dependent on pH and ionic strength of water to be treated. Donnan dialysis pilot was equipped with specific anion exchange membranes to reduce electrolyte leakage and thus increased in mineralization of treated water. By this treatment cation composition of treated water was not modified but all the anion except chloride was partially eliminated and substituted chloride ions.

The use of magnesium titanate as an adsorbent for fluoride removal was examined [73]. The amount of fluoride adsorbed from 4 mgL^-1^ of fluoride solution was found to be 0.029 mg/g. The influence of different parameters such as initial concentration of adsorbent, adsorbent dose, agitation time, co-ions and temperature on defluoridation was studied. The percentage of fluoride removal increased with increase in initial concentration of fluoride, temperature, and adsorbent dose and agitation time up to 40 min. Wide range of pH and high temperature ranges were found as the optimum conditions for fluoride adsorption. The experimental data fitted satisfactorily (r > 0.97) to Langmuir isotherm. Thermodynamic parameters such as ΔHº, ΔSº and ΔGº concluded the adsorption was endothermic. Moreover the mechanism of adsorption was found to be physisorption from the magnitude of enthalpy change 20–45 KJ/mol. Defluoridation of water using bauxite adsorbent was studied [74]. The optimum adsorbent dose was found to be 5 g/100 mL, the equilibrium contact time was found to be 75 min and maximum adsorption obtained at pH 6. Maximum fluoride removal was found to be 94.2% at optimum conditions. Langmuir isotherm fitted well for defluoridation of water using bauxite.

In very recent study, bauxite as an adsorbent for the removal of fluoride from contaminated ground water was used. Adsorption experiments with respect to variation in time, pH, adsorbate and concentrations of other anions namely nitrate, sulfate, carbonate, and phosphate were carried out. To get a better insight into the mechanism of adsorption they were characterized bauxite before and after fluoride adsorption by XRD, FTIR and SEM–EDX. An adsorption rate was rapid and followed first order kinetics with intraparticle diffusion as the rate determining step. They were also estimated thermodynamic parameters (ΔH°, ΔS° and ΔG°) which are indicating that the adsorption was spontaneous and exothermic in nature [75]. Mn-Ce oxide adsorbent by co-precipitation method was prepared [76] and studied the role of prepared adsorbent in fluoride removal from the sorption isotherms. It has been shown that the maximum sorption capacities of fluoride on the powdered and granular Mn-Ce adsorbent were 137.5 and 103.1 mg/g.

In our lab, we are also synthesizing Mn-Zr, Mn-Ce-Zr, Mn-Ti-Ce etc. as nano adsorbent with high sorption capacity for fluoride removal from potable water. In this study, the sorption isotherms showed that the maximum adsorption capacities of fluoride on the powdered and granular Mn-Zr adsorbent. The sorption experiment was carried out in 250 mL polypropylene flask containing 200 mL of fluoride solution and 0.02 g adsorbent, and flask were shaken at 150 rpm in a shaker at 25°C for 24 h. The adsorption experiment was carried out at the initial fluoride concentration of 10 mg/L while after treatment water has fluoride concentration in range of 5–7 mg/L. The concentration of fluoride in treated water was varied with contact time, concentration of adsorbent, pH, and concentration of fluoride in non-treated water. The individual particle size, as determined by TEM and XRD and the specific surface area of each sample. There is two size ranges as determined by the TEM images given for the samples. This is due to the fact that the Mn-Zr samples possess a very wide size distribution. In real, there is no discreet break between the large and small particles in these samples. The large and small particles are separated here because important information about the exposed surface planes can be determined by examining the large particles while the small particles are too small to view anything of value.

The paper presents a detailed study of the effect of manganese dioxide on the defluoridation potential of disposed earthenware (DEW) of the particle size less than 300 μm. Manganese dioxide was added to DEW with weight content from 0.01 to 0.025%. The defluoridation was investigated in static experiments, at pH 5–11 and with contact time of 35 min. The fluoride removal increased with the increasing content of manganese dioxide. In static sorption, the defluoridation with DEW dispersed with 0.025% of manganese dioxide increased from 1198 to 1888 mg/kg when the pH increased from 5 to 7 [77]. In the simulating equilibrium data, simple kinetic models namely, pseudo I and II order, particle and pore diffusion, Elovich and isothermal models of Langmuir and Freundlich were used. The fluoride removal was investigated in the presence of coexisting ions. It was found that the reduction in fluoride sorption was greater in the presence of ${\mathrm{SO}}_{4}^{2-}$ ion than in the presence of ${\mathrm{HCO}}_{3}^{-}$, Cl^−^ and ${\mathrm{NO}}_{3}^{-}$. DEW with dispersed manganese dioxide, showed an ability to lower the fluoride concentration to acceptable levels and improved the defluoridation efficiency of unmodified DEW. The spent sorbent was easily regenerated by NaOH solution. See Table 5 for details.

**Table 5:** **Adsorption capacities** (**AC**) **and other parameters for the removal of fluoride by metal oxides**/**hydroxides**/**oxyhydroxides**, **mixed metal oxides**, **metal**- **impregnated oxides as adsorbents**

**Adsorbent**	**AC (mg/g)**	**CR (mg/L)**	**pH**	**Ref.**
Magnesia-amended silicon dioxide	12.6	-	3.0	[64]
Superparamagnetic sorbent(ZrO_2_/SiO_2_/Fe_3_O_4_)	14.7	10-105	4	[65]
Calcined meixnerite	16.0	12.4-248	6.5	[66]
Al-Ce hybrid adsorbent	91.4	2-15	6.0	[67]
Aluminium titanate, Bismuth aluminate	3.01, 7.09	2-10	7.0	[69]
Magnesia, Magnesia/chitosan composite	2.2, 4.4	10-23	10-10	[70]
Magnesium Titanate	0.03	2-10	3-11	[73]
Bauxite	5.16	4-26	6	[74]
Mn-Ce oxide adsorbent powdered & granular	79.5 & 45.5	10-100	6-8	[76]
Manganese dioxide with earthenware	9.02	3-6	7.15	[77]

#### Bio adsorbents

The defluoridation capability of brushite-calcite with two local biosorbents material (grind neem and pipal leaves) was compared [78]. Fluoride concentration was found to be reduced from 5 mg/L to 1.2 mg/L in 90 min and decreased to 1 mg/L in 18 h by brushite calcite while local biosorbents materials reduced the fluoride concentration to 4 mg/L in 90 min and to 3.22 mg/L after 18 h thus indicating the superiority of brushite-calcite to biosorbents (neem and papal leaves). The Cynodon dactylon was prepared from the activated carbon for fluoride removal [79]. The fluoride concentration of 3 mg/L with 1.25 g of adsorbent at neutral pH was found to be removed to 83.77%. Adsorbent was regenerated by 67.4% using 2% NaOH. Fluoride removal was hindered by bicarbonate ions. The sorption of fluoride was found to be spontaneous and endothermic following pseudo-second order kinetics.

The applicability of neodymium-modified chitosan as adsorbents for the removal of excess fluoride from water was investigated [80]. The modified chitosan showed defluoridation capacity at pH 7. The defluoridation capacity increased with increasing temperature which indicated the strong tendency of monolayer formation process to occur. The chloride, sulphate and nitrate showed no significant effect within the concentration range tested. The Langmuir maximum equilibrium sorption was found to be 11.411 - 22.38 mg/g at different temperatures. The defluoridation capacity of chitosan beads was found negligible and it was chemically modified by introducing the multifunctional groups, such as ammonium and carboxylic groups by mean of protonation and carboxylation in order to utilize both amine and hydroxyl groups for fluoride removal. That modified bioadsorbent showed maximum defluoridation capacity at pH 7. The defluoridation capacity of protonated cum carboxylated chitosan beads was found (1800 mg/F^−^/kg), which was higher than raw chitosan beads (52 mg/F^−^/kg). The protonated cum carboxylated chitosan beads removed fluoride by hydrogen bonding [81]. To remove fluoride ions from aqueous solutions eco-friendly conducting polymer/bio-polymer composites viz. polyaniline/chitosan (PANi/Ch) and polypyrrole/chitosan (PPy/Ch) as adsorbents were investigated. Chitosan is one of the promising natural polymers with characteristics such as biodegradability, chemical inertness, good film forming properties and low cost. The system variables studied include initial concentration of the sorbate, agitation time, adsorbent dose, pH, co-ions and temperature. At low pH and high temperature, fluoride removal occurred. The amount of fluoride ion removal increased with a decrease in adsorbent dose due to the availability of higher number of fluoride ions per unit mass of polymer composites, i.e., higher fluoride/composite ratio. According to Langmuir and Freundlich isotherms, the experimental data fitted well. The amounts of fluoride ions adsorbed per unit mass of the adsorbents were found to be 5.9 mg/g for PANi/Ch and 6.7 for PPy/Ch, at 50°C from 10 mg/L fluoride solution. The removal of fluoride ions is an endothermic process was indicated through thermodynamic parameters. The PPy/Ch composite exhibited relatively higher defluoridation capacity than the PANi/Ch composite due to the fact that polypyrrole itself exhibited higher fluoride ions removal capacity than that of polyaniline [82]. Therefore, the corresponding polymer/Ch composites also exhibited the same trend. It is well established that these polymers, in the chloride ion doped form, remove fluoride ions from water via dopant-exchange mechanism. The conducting polymer/Ch composites remove fluoride ions from water through the ion-exchange mechanism using the N-atoms present in both the constituent polymers and this ion- exchange leading to an enhanced fluoride removal. The defluoridation occurred through dopant-exchange mechanism on the N-atoms present in these constituent polymers.

To enhance the fluoride removal capacity from water, Lanthanum incorporated chitosan beads were prepared using precipitation method. At pH 5, maximum adsorption capacity was observed 4.7 mg/g [83]. Waste fungal biomass [84] (Pleuratus astreatus 1804) derived from the laccare fermentation process was used for fluoride removal from water. Batch absorption studies were performed for this experiment and the results revealed that bioadsorbent demonstrated the ability to absorb fluoride from water. The sorption obeyed the pseudo-first-order rate equation and the fluoride sorption was found to be dependent on the aqueous phase pH. At lower pH, the fluoride uptake was observed to be greater. A detailed fluoride adsorption study in packed columns with chitin or a chitin-based biocomposite is reported [85]. A novel cost effective defluoridation method that is based on surface modification of rice husk ash (RHA) by coating aluminum hydroxide [86]. See Table 6 for details.

**Table 6:** **Adsorption capacities** (**AC**) **and other parameters for the removal of fluoride by Bio adsorbents**

**Adsorbent**	**AC (mg/g)**	**CR (mg/L)**	**pH**	**Ref.**
Neodymium-modified chitosan	22.4	10-100	7.0	[81]
PANi/Ch, PPy/Ch	5.9, 6.7	2-10	7.0	[82]
Lanthanum incorporated chitosan beads	4.7	5.34	5.0	[83]
Chitin	3.4	1-100	4.8	[85]
Rice husk ash (RHA) by coating Al(OH)_3_	9-10	10-60	5	[86]

#### Carbon based sorbents

Some researchers used carbon as an adsorbent for fluoride removal. The potential sorption capacity of multi-walled carbon nanotubes (MWCNTs) was investigated as a means of removing fluoride from the drinking water of a number of regions in Iran and from experimental solutions [87]. A novel poly(aniline-co-o-aminophenol) (PAOA) modified carbon felt electrode reactor was designed and investigated for fluoride removal from aqueous solutions [88]. Fishbone charcoal is a moving media adsorption system used for fluoride adsorption [89]. The ratio of attained of attained equilibrium sorbate concentration to the initial sorbate concentration and the fluoride removal capacity of the sorbent were found to vary inversely with the sorbent mass input rate and varied directly with the sorbate flow rate and initial sorbate concentration. The ratio of attained equilibrium sorbate concentration to the initial sorbate concentration found to be a function of the sorbent – sorbent mass input rate ratio. Zirconium impregnated activated charcoals as an adsorbent used for defluoridation [90]. In comparison of plain activated charcoal, the fluoride adsorption capacity of impregnated activated charcoals was 3–5 times higher. Maximum fluoride uptake showed by zirconium impregnated coconut fibre charcoal and followed by groundnut shell and coconut shell charcoals due to its large surface area. Micro/nano-hierarchal web consisting of activated carbon fibres [91] and carbon nano fibres impregnated with Al used as an adsorbent for fluoride removal from wastewater. At pH 5–8, Al-carbon nano fibres was used for treating the wastewater. Granular activated carbon was coated with manganese oxides and used for fluoride removal from water and showed maximum adsorption capacity [92]. The adsorption capacity was three times higher than uncoated granular activated carbon. At pH 3, the fluoride adsorption was observed maximum. Different grades of graphite were used as adsorbents for fluoride removal from water [93]. At appreciable extent, competing anions did not affect fluoride removal. Carbons loaded with specific chemical moieties were prepared from pecan nut shells employing a natural modifier agent obtained from egg shell (CMPNS), which is rich in calcium, for the selective adsorption of fluoride from water [94]. A batch adsorption system was applied to investigate the adsorption of fluoride from aqueous solution by graphene [95]. To the removal of excess fluoride in drinking water using cerium dispersed in carbon (CeDC), a hybrid sorbent which was prepared by carbonization of ammonium cerium sulphate impregnated starch [96].

The importance of *Acacia Arabica* fruit carbon as an alternative to activated carbon as defluoridation method was investigated [97]. It was concluded that, for the selected domain, the chosen variables agitation time (T), granulometry (G) and adsorbent’s concentration (A) had very significant influence on the process, with increasing importance as followed: G < T < A. The role of three activated carbon adsorbents, BKC, BOC and RSC which were prepared from biomaterials of bergera koenigh (curry leaf seeds), batavia orange and raphanus sativus (garden radish) respectively was investigated [98]. It has been found that upto 4 mg/L fluoride contents can be reduced to permissible level. Maximum contact time was found to be 30 min and optimum dose of adsorbent was 1 g/L. Moreover, at pH 6 maximum defluoridation was observed. Adsorbent was found to be regenerated by passing 0.1 M NaOH solutions. See Table 7 for details.

**Table 7:** **Adsorption capacities** (**AC**) **and other parameters for the removal of fluoride by Carbon based sorbents**

**Adsorbent**	**AC (mg/g)**	**CR (mg/L)**	**pH**	**Ref.**
MWCNTs	-	1-100	5-9	[87]
(PAOA) modified carbon	3.2-11.8	50-200	7	[88]
Al-impregnated hierarchal web of carbon fibres	17.0	0.1-50	-	[91]
MnO_2_-coated granular activated carbon	-	3-35	5.2	[92]
Various grades of graphite	0.16-3.13	2-10	7.0	[93]
CMPNS	2.3	5–40	7.0	[94]
Graphene	17.65	5-30	7.0	[95]
cerium dispersed in carbon (CeDC)	52	2.8-8.3	8.0	[96]

#### Natural materials

Some researchers have been used naturally occurring adsorbent for fluoride removal from water due to its low cost availability. The three different coal based sorbents, lignite, fine coke and bituminous coal was used for fluoride removal [99]. At acidic pH, fine coke and bituminous coal showed higher fluoride removal from water. The pH range 6–12 was found favourable for fluoride removal from water in case of lignite. The capacities of coal based adsorbents ranged between 6.9 and 7.44 mg/g. Assam coals were used for fluoride removal by researchers [100]. For 85% fluoride removal, the optimum dose of adsorbent was found to be 1.25 g/100 mL. The fluoride adsorption affected through particle size. Fired clay chips used for fluoride removal [101]. At pH 3 and 9, the maximum amount of fluoride removal after equilibration was 90% and 80%, respectively. South African clays used for fluoride removal from water [102]. For fluoride adsorption, various clay such as bauxite, laterite, palygorskite, bentonite and kaolinite were tested. Clays consisting of substantial amounts of gibbsite or aluminium oxides exhibited best overall potential as fluoride adsorbents. The defluoridation with three types of tamarind seed, pristine (PriTS), purified (PurTS) and polyaniline (Pani) was reported [103]. It has been found that 50% pani coated both in pristine and purified TS materials enhanced the fluoride adsorption efficiency. The defluoridation of water using tamarind seed by domestic water filter was studied [104]. The influence of pH, agitation time, initial fluoride concentration, temperature, particle size and solvent dose were studied for defluoridation. Maximum defluoridation capacity was achieved at pH 7. Tamarindus indica fruit shells (TIFSs) were activated by ammonium carbonate and then carbonized leading to carbon abbreviated as ACA–TIFSC [105]. The defluoridation capacity decreased with increase in initial fluoride concentration, temperature and particle size. Defluoridation followed first order kinetics and Langmuir adsorption isotherm. The hydro-methanolic extract of tamarind fruit pulp in removing of body fluoride burden has been undertaken for study [106]. For this experiment thirty rats were divided into five groups. Low dose, middle dose, high dose of sodium fluoride was received by these groups through orally at the rate of 200 mg/kg body weight daily for four weeks. Rats of low dose, middle dose and high dose group simultaneously received tamarind fruit pulp extract at three doses 25 (low), 50 (medium) and 100 mg (high) per kg body weight orally, respectively. The concentration of fluoride in blood, urine and long bone of experimental rats was monitored to assess the efficacy of the extract. The mean serum fluoride concentration in fluoride exposed rats was 0.145 ± 0.009 and0.783 ± 0.042 g/mL on days 0 and 98. In comparison, fluoride concentrations in tamarind treated rats were 0.179 ± 0.021 and 0.633 ± 0.015; 0.179 ± 0.021 and 0.502 ± 0.025 and 0.176 ± 0.021 and 0.498 ± 0.030 g/mL in low, medium and high dose groups, respectively on day 0 and day 98 of the experiment. There was a significant increase in urinary fluoride excretion from day 28 onwards. The mean fluoride concentration in long bones of treated rats was significantly lower than the values recorded from fluoride exposed rats. Adsorption was carried out by pumice stone [107] as an adsorbent for fluoride removal. See Table 8 for details.

**Table 8:** **Adsorption capacities** (**AC**) **and other parameters for the removal of fluoride by Natural materials**

**Adsorbent**	**AC (mg/g)**	**CR (mg/L)**	**pH**	**Ref.**
Lignite, Fine coke and Bituminous coal	6.9-7.4	3-90	5-10	[99]
Fired clay chips	7.2-9.0	5-20	5-7	[101]
Tamarindus indica Fruit Shells (TIFSs)	22.3	2-8	7	[105]
Pumice stone	41	10	3-10	[107]

#### Nano-sorbents

The potential of nano alumina for fluoride removal and was found to be 140 mg/g [108]. Defluoridation studies were conducted under various experimental conditions such as pH, contact time, initial fluoride concentration, temperature and the presence of counter ions. It was noticed that maximum fluoride removal occurred at pH 6.15 and increased with increase in time and initial fluoride concentration. Fluoride adsorption was not significantly affected by temperature variation but was influenced by ${\mathrm{PO}}_{4}^{3-}$, ${\mathrm{SO}}_{4}^{2-}$ and ${\mathrm{CO}}_{3}^{2-}$ ions. The sorption isotherm was fitted with Langmuir model and followed pseudo-second order kinetics. Carbon nanotubes (CNTs) as support to deposit Al_2_O_3_ and explored the possibility of Al_2_O_3_/CNTs for fluoride removal from drinking water [109]. The fluoride removal was occurred on Al_2_O_3_/CNTs at pH 5.9-9.0. The adsorption capacity for Al_2_O_3_/CNTs was 13.5 times higher than AC-300 carbon and four times higher than that of γ-Al_2_O_3_ at equilibrium fluoride concentration of 12 mg/L. The mass of fluoride adsorption for Al_2_O_3_/CNTs at pH 6 reached 28.7 mg/g at equilibrium concentration of 50 mg/L. See Table 9 for details.

**Table 9:** **Adsorption capacities** (**AC**) **and other parameters for the removal of fluoride by Nano**-**sorbents**

**Adsorbent**	**AC (mg/g)**	**CR (mg/L)**	**pH**	**Ref.**
Nano – alumina	14.0	1-100	6.15	[108]
Al_2_O_3_/CNTs	28.7	50	6.0	[109]

#### Building materials

The potential of building materials towards fluoride removal was observed. The low weight concrete (building material) [110] as an adsorbent for the removal of fluoride from water was used and check their efficacy by vary various parameters. The maximum adsorption of fluoride took place at pH 6.9 but in the acidic medium, less adsorption took place due to the formation of weakly ionised hydrofluoric acid. Another adsorbent i.e. hydrated cement [111] and hardened alumina cement granules [112] for fluoride removal from aqueous solution was observed. See Table 10 for details.

**Table 10:** **Adsorption capacities** (**AC**) **and other parameters for the removal of fluoride by Building materials**

**Adsorbent**	**AC (mg/g)**	**AD (mg/g)**	**CR (mg/L)**	**pH**	**Ref.**
LWC (building material)	5.15	0.10	-	6.9	[110]
Hydrated cement	2.7	1000	-	6.7	[111]
Hardened alumina cement granules	34.4	10.2	2.5-100	-	[112]

#### Apatite and hydroxyapatite

Apatite in different forms has been used for fluoride removal as it showed good prospective for defluoridation. Synthetic nano-hydroxyapatite (n-Hap), biogenic apatite, treated biogenic apatite, geogenic apatite were engaged to evaluate their effectiveness for fluoride removal [113]. The removal of fluoride using synthetic hydroxyapatites (HAps) was investigated. It was found that small sized (HAps) were more efficient than the largest particle size. It was also concluded that the defluoridation efficiency increased with increase in the dose of HAps and contact time but decreased with increase in initial fluoride concentration and pH. The nanostructure of hydroxyapatite from combined ultrasonic and microwave technique and examined its role in defluoridation [114,115]. The effects of low molecular weight organic acids (LMWOAs) on the defluoridation capacity of nanosized hydrpxyapatite (nHAP) from aqueous solution were investigated [116]. Cellulose@hydroxyapatite (HA) nanocomposites were prepared in NaOH/thiourea/urea/H_2_O solution via situ hybridization [117]. Aluminum-modified hydroxyapatite (Al-HAP) was also used for defluoridation [118]. Phosphogypsum (PG) was utilized to prepare hydroxyapatite nanoparticles with high purity. nHAP derived from PG exhibits excellent adsorption capacity for fluoride [119]. See Table 11 for details.

**Table 11:** **Adsorption capacities** (**AC**) **and other parameters for the removal of fluoride by Apatite and hydroxyapatite**

**Adsorbent**	**AC (mg/g)**	**AD (g/L)**	**CR (mg/L)**	**pH**	**Ref.**
Synthetic nano hydroxyapatite	4.575	4	3-80	5-6	[113]
Biogenic apatite	4.99				
Treated biogenic apatite	6.849				
Geogenic apatite	0.014				
Hydroxyapatite	1.432	20	3-80	5	[114]
Nano sized hydroxyapatite	3.44	0.2	50	5	[116]
Cellulose@hydroxyapatite nanocomposites	4.2	4	10	6.5	[117]
Al-modified hydroxyapatite (Al-HAP)	32.57	0.5	5-50	7	[118]
nHAp derived from PG	19.74-40.81	1	10-50	2-11	[119]

#### Industrial waste adsorbent

Extensive industrial actions generates enormous amount of solid waste materials as by–products. One of the advantageous uses of these wastes is to convert them as economical sorbents for detoxification of water. For the fluoride removal from aqueous solution, the industrial waste like spent bleaching earth (SBE) is used as a adsorbent [120]. The waste SBE was obtained from the oil industry, and acid and alkaline were used to recover it. In addition, the optimum conditions obtained in this study were tested on Kuhbonan (one of the regions of Iran whose fluoride level has been reported between 2.28 and 5.4 mg/L) water. At pH 7, the maximum fluoride adsorption was obtained and an equivalent time of 180 min. When the fluoride initial concentration in water increased, fluoride removal efficiency decreased. The maximum adsorption capacity of RSBE was 0.6 mg/g fluoride (2.5–8 mg/F^−^/L, 10 g RSBE/L and pH 7). Adsorption of fluoride on waste carbon slurry (a fertilizer industry waste) was investigated [121]. Activated titanium rich bauxite was also another adsorbent which was used for defluoridation of water [122]. See Table 12 for details.

**Table 12:** **Adsorption capacities** (**AC**) **and other parameters for the removal of fluoride by Industrial waste adsorbent**

**Adsorbent**	**AC (mg/g)**	**AD (g/L)**	**CR (mg/L)**	**pH**	**Ref.**
Regenerated spent bleaching earth	0.6	10	2.5–8	7.0	[120]
Waste carbon slurry	4.3	1	1-11	7.6	[121]
Activated titanium rich bauxite	3.7-4.1	-	2-50	6.0	[122]

#### Zirconium based adsorbents

The defluoridation performance and adsorption mechanism of a high capacity hydrous zirconium oxide [123], meso-structured zirconium phosphate (MZrP) [124] adsorbents were investigated. The need of treatment of large volume water samples could be met by the super paramagnetic properties of the nanoparticles through application of an external magnetic field. Zr(IV) metalloporphyrins possess high selective affinity toward fluoride. The synthesis of a new sorbent consisting of 3-aminopropyl triethoxysilane (APTES) coated magnetic nanoparticles functionalized with a zirconium(IV) porphyrin complex Zr(TCPP)Cl2 [TCPP: tetrakis(4-carboxyphenyl) porphyrin] [125] were used for defluoridation. A novel zirconium(IV)-ethylenediamine (ZrEDA) hybrid material was prepared by mixing aqueous solution of zirconium oxychloride (0.1 M) and aqueous solution of ethylenediamine (0.1 M) following an environmental friendly sol–gel method [126]. zirconium-modified-Na-attapulgite (Zr-A) adsorbent was used for defluoridation [127]. See Table 13 for details.

**Table 13:** **Adsorption capacities** (**AC**) **and other parameters for the removal of fluoride by Zirconium based adsorbents**

**Adsorbent**	**AC (mg/g)**	**CR (mg/L)**	**pH**	**Ref.**
Hydrous zirconium oxide adsorbent	124 -68	2-120	4-7	[123]
Meso-structured zirconium phosphate (MZrP)	-	1-10	6	[124]
Zr(IV)metalloporphyrin grafted Fe_3_O_4_ NPs	-	1-10	5.5	[125]
Zr(IV)–ethylenediamine	37.03	2-50	7	[126]
Zr-modified-Na-attapulgite(Zr-A)	24.55	10-50	3.7–7.5	[127]

#### Miscellaneous adsorbents for defluoridation of water

Bleaching powder as adsorbent for fluoride removal from water was used. It could be used as disinfectant and defluoridation agent. It was found that defluoridation from water occurred due to adsorption by bleaching powder and slightly due to precipitation in the form of calcium fluoride. At pH 6–10, defluoridation from water was occurred by adsorbent dose of 50 g/L [128]. Ti-Ce (9.6 mg/g) and Ti-La (15.1 mg/g) hybrid adsorbents had higher sorption capacities for fluoride than TiO_2_ (1.7 mg/g) adsorbent [129]. The sorption capacity decreased with increasing pH from 3 to 9.5. The conducting polypyrrole as adsorbent for the fluoride removal from aqueous solution was studied [130]. The amount of fluoride ion removed per unit mass of the adsorbent at 30°C from 10 mg/L fluoride ion solution was estimated to be 6.37 mg/g. For defluoridation from aqueous solution, Magnesia-loaded fly ash cenospheres was prepared by wet impregnation of fly ash cenospheres with magnesium chloride solution. At pH 3, defluoridation from aqueous solution was observed with adsorbent dose 2.5 mg/L [131]. Aerobic granules (AG) were carboxylated and Ce(III) was incorporated to obtain modified granules (Ce(III)–MAG) for removal of fluoride from aqueous solutions [132]. Besides the above mentioned adsorbents, various other authors also examined the potential of different types of sorbents such as KMnO_4_ modified activated carbon derived from steam pyrolysis of rice straw [133], hybrid thorium phosphate composite [134], granular acid-treated bentonite [135], Nickel and magnesium hydrotalcite-like compounds (NiAlHT, MgAlHT) [136], etc. for fluoride removal. See Table 14 for details.

**Table 14:** **Adsorption capacities** (**AC**) **and other parameters for the removal of fluoride by miscellaneous adsorbents**

**Adsorbent**	**AC (mg/g)**	**CR (mg/L)**	**pH**	**Ref.**
Bleaching powder	0.2	5	6.7	[128]
Magnesia loaded fly ash cenospheres	6.0	100	3.0	[131]
Modified granules (Ce(III)–MAG)	4.80	4-20	3-5	[132]
KMnO_4_ modified carbon	15.9	20	2.0	[133]

## Conclusions

Studies for the removal of fluoride, using several adsorbents have been summarized briefly in this review. The efficacy of each adsorbent has been examined and discussed. The following conclusions have been made on the basis of literature review:

•Although activated alumina adsorption technology shows higher uptake of fluoride, but it is expensive and its performance is affected by the presence of co-ions in water.

•Rare earth oxide-based materials have shown high fluoride removal efficiency in batch mode but these materials have been found very expensive.

•Carbon based adsorbents have its application in small scale and lack in terms of column operation and/or pilot scale.

•Various natural adsorbents have potential for defluoridation of water but their difficulties in regeneration and low efficiency have also been reported.

•Biosorption is an environmentally friendly technique for fluoride removal utilizing various biomaterials of low cost. However, there are some disadvantages also, which limited its use for removal of low fluoride concentration.

•Nano- adsorbents have been attracted considerable attention in the recent years in fluoride removal and these materials have shown higher fluoride uptake capacity.

•The influence of pH, agitation time, initial fluoride concentration, temperature, particle size, surface area, presence and nature of counter ions and solvent dose were studied for defluoridation with various adsorbents.

•The sorption kinetics was pseudo-second order or pseudo-first order and the equilibrium data fitted well to the adsorption isotherms like Langmuir and Freundlich.

So, the future research should be concentrated in evaluating the efficacy of adsorbents in terms of cost and feasibility for removal of fluoride. It would be worthwhile to study the suitability of different chemicals to regenerates the spent adsorbents.

## Abbreviations

AA: Activated Alumina; MGA: Metallurgical grade alumina; UHA: Untreated hydrated alumina; THA: Thermally treated hydrated alumina; PPy: Polypyrrole; Fe(III)-STI: Fe(III)-stilbite zeolite; AFM: Atomic force microscopy; XPS: X-ray photoelectron spectroscopy; SEM: Scanning electron microscopy; FTIR: Foutier infrared spectroscopy; XRD: X-ray diffraction; CA: Calcium aluminate; SO4: Titanium oxysulfate TiO(SO4); AT: Aluminium titanate; BA: Bismuth aluminate; MgOC composite: Magnesia (MgO) and magnesia/chitosan; enthalpy ΔH0 and entropy ΔS0: Gibbs free energy ΔG^0^; cMgOP: Calcined magnesia with pullulan; DEW: Disposed earthenware; PPy/Ch: Polyaniline/chitosan (PANi/Ch) and polypyrrole/chitosan; RHA: Rice husk ash; MWCNTs: Multi-walled carbon nanotubes; PAOA: Poly(aniline-co-o-aminophenol); CMPNS: Carbons loaded with specific chemical moieties pecan nut shells; CeDC: Cerium dispersed in carbon; T: Agitation time; G: Granulometry; A: Adsorbent’s concentration; BKC: Bergera koenigh (curry leaf seeds); BOC: Batavia orange; RSC: Raphanus sativus (garden radish); (PriTS: Tamarind seed, pristine; PurTS: Purified; Pani: Polyaniline; TIFSs: Tamarindus indica Fruit Shells; ACA–TIFSC: Ammonium carbonate Tamarindus indica Fruit Shells carbon; CNTs: Carbon nanotubes; LWC: Low weight concrete; n-Hap: Synthetic nano-hydroxyapatite; LMWOAs: Low molecular weight organic acids; Al-HAP: Aluminum-modified hydroxyapatite; PG: Phosphogypsum; SBE: Spent bleaching earth; RSBE: Regenerated spent bleaching earth; MZrP: Meso-structured zirconium phosphate; APTES: 3-aminopropyl triethoxysilane; TCPP: Tetrakis(4-carboxyphenyl) porphyrin; ZrEDA: Zirconium(IV)-ethylenediamine; Zr-A: Zirconium-modified-Na-attapulgite; AG: Aerobic granules; NiAlHT: MgAlHT, Nickel and magnesium hydrotalcite-like compounds; Ce(III)–MAG: Ce(III) modified granules; AC: Adsorption capacity; CR: Concentration range; AD: Adsorbent dose

## Competing interests

Both the authors declared that there is no competing interest.

## Authors’ contributions

VT and DK both authors made equally efforts in this paper and have created many of the chemistry related stuffs in this review. Both authors read and approved the final manuscript.
